# Effect of Early Rehabilitation Nursing on Motor Function and Living Ability of Patients with Traumatic Brain Injury Based on Orem's Self-Care Theory

**DOI:** 10.1155/2022/7727085

**Published:** 2022-09-08

**Authors:** Yuqin Yang, Lu Niu

**Affiliations:** The First People's Hospital of Lianyungang, The Affiliated Lianyungang Hospital of Xuzhou Medical University, The First Affiliated Hospital of Kangda College of Nanjing Medical University, Nanjing 222061, China

## Abstract

**Objective:**

To explore the effect of early rehabilitation nursing on motor function and living ability of patients with traumatic brain injury (TBI) based on Orem's self-care theory.

**Methods:**

A total of 60 patients with TBI treated in our hospital from February 2019 to June 2021 were enrolled. The patients were randomly divided into a control group and a research group. The control group adopted the early rehabilitation nursing model, while the research group adopted the early rehabilitation nursing model based on Orem's self-nursing theory. Nursing satisfaction, Fugl-Meyer score, NIH-SS score, Barthel index, quality of life score, and compliance were in the comparison of the two groups.

**Results:**

The nursing satisfaction of the research group was higher than that of the control group (*P* < 0.05). Compared with the control group, the Fugl-Meyer scores of the research group were higher at 1 month, 2 months, and 3 months after nursing (*P* < 0.05). After nursing, the NIH-SS score of the two groups decreased. In the comparison of the two groups, the NIH-SS score of the research group at 1 month, 2 months, and 3 months after nursing was lower (*P* < 0.05). After nursing, the Barthel index of the two groups increased. In the comparison of the two groups, the Barthel index of the research group was higher compared to the control group at 1 month, 2 months, and 3 months after nursing (*P* < 0.05). The scores of physiological function, psychological function, social function, and health self-cognition in the research group were lower (*P* < 0.05). The compliance rate of the research group was higher than that of the control group (*P* < 0.05).

**Conclusion:**

Patients with TBI receive early rehabilitation nursing based on Orem's self-care theory, which can effectively improve patient satisfaction and compliance and achieve the purpose of improving motor function and living ability. This nursing program is worth popularizing in the clinic.

## 1. Introduction

Brain injury, also known as traumatic brain injury (TBI) or head injury (HI), refers to brain injury caused by external violence in the head and neck, which can lead to disturbance of consciousness, memory loss, and neurological dysfunction [1]. TBI is a kind of injury with high morbidity, high mortality, and high disability rate. The prevalence rate of TBI in China is 783.3/100,000 population, second only to cerebrovascular disease and limb injury. However, the mortality and disability rate ranked first [2]. In the United States, the incidence of TBI is about 200/100,000 people; there are 500,000 new cases every year, and about 80,000 people die from TBI each year [3]. The fatality rate of mild, moderate, and severe TBI is 0.7%, 26% and 58%, respectively. The disability rate is 10%, 66%, and 100%, respectively [4]. The main causes of TBI include traffic accidents, industrial accidents, accidental falls, sports injuries, falls, dystocia, and surgical delivery. Wounds such as gunshot wounds and explosive wounds, as well as car accidents, fortifications, and building collapses, are the main causes of TBI in wartime [5]. TBI can occur in all age groups, and its distribution is polarized, that is, teenagers aged 15–24 (200/100,000 population) and people aged 65–75 (200/100,000 population). The incidence of TBI in males was significantly higher compared to females, about 2:1, and the mortality rate was 3–4 times higher compared to females [6]. TBI occurs in youth and early adulthood, which are two critical periods in life. During this period, people not only need to learn knowledge but also need to master the skills for future life, but TBI limits their learning and ability development. Even have a significant impact on independent life [7]. TBI is the most common and crippling disease of the nervous system, which is the most disabling qualitative disease that can occur in patients with different degrees of motor and sensory dysfunction, with cognition and perception function, communication function, daily life self-care ability, behavior, psychological and social barriers; these dysfunction leads to higher morbidity. It seriously affects the quality of life of patients and brings a huge impact and heavy burden on patients, families, and society. In recent years, with the progress of medical technology, the overall mortality rate of brain trauma has dropped from 50% 30 years ago to about 30% now, but most of the surviving patients will be left with varying degrees of disability. In addition to physical and language dysfunction, cognitive impairment accounted for a large proportion [8].

Surgery is commonly employed in the treatment of TBI, but how to effectively providing nursing services for patients plays an important role in promoting postoperative rehabilitation and enhancing the quality of life of patients [9]. In order to explore the clinical nursing model suitable for TBI, further meet people's continuous pursuit of health, maximize the psychological and physiological satisfaction of patients with TBI, and reduce the burden on patients' families, nurses, and society. In recent years, major medical institutions have introduced self-care theory into nursing work [10]. Self-care theory is a nursing theory put forward by Dorothea Elizabeth Orem, Master of Nursing Education in the United States [11]. It was introduced into China in the middle and late 1980s, then gradually recognized by the nursing community, and applied to clinical practice guidance, education, and research. It includes self-care theory, self-care defect theory, and nursing system theory, which is composed of the nursing behavior provided by nurses and the behavior of patients. Nurses adopt three different nursing systems according to patients' self-care needs and ability, namely, complete compensation system, partial compensation system, and support-education system. It is widely employed in the nursing care of patients with TBI. In full compensation care, nurses take care of elderly TBI patients to meet all their needs. Nurses must make every effort for these patients to meet their therapeutic self-care needs. In partial compensatory nursing, both nurses and patients with TBI can play an important role in meeting the needs of therapeutic self-care. Nurses “help” patients complete self-care activities and make up for the deficiency of patients' self-care. According to the needs of patients, they can help and adjust their self-care ability, while patients try their best to complete what they can do independently, adjust their self-care ability, meet their self-care needs, and accept nurses' help. In the support-education system, patients with TBI need to learn how to self-care and the patients are able to complete self-care activities but need temporary help. The help provided by nurses is psychological support, technical guidance, and the provision of a needed environment. In this system, the responsibility of a nurse is to “do it for him” and “do it for him” in the first two systems and transition to “educate and support him to do it.” Early rehabilitation exercise refers to timely and correctly guiding patients to apply exercise therapy on the basis of a comprehensive evaluation of patients' postoperative physical function according to the principles of different people, gradual and orderly progress, perseverance, dynamic and static combination, and active participation [11]. The purpose of early rehabilitation exercise is to enhance muscle strength, increase the range of joint motion, and restore the function of lumbar vertebrae and lower extremities. The early rehabilitation nursing model based on Orem's self-care theory is to carry out early rehabilitation nursing for patients under Orem's self-care theory, in order to promote the recovery of motor function and living ability [12]. This study focuses on the effect of early rehabilitation nursing based on Orem's self-care theory on motor function and living ability of patients with TBI.

## 2. Patients and Methods

### 2.1. General Information

A total of 60 patients with TBI treated in our hospital from February 2019 to June 2021 were enrolled. The patients were randomly divided into a control group and a research group. The control group adopted the early rehabilitation nursing model, while the research group adopted the early rehabilitation nursing model based on Orem's self-nursing theory. In the control group, the age was 35–78 years old, with an average of (50.91 ± 3.53) years, including 15 males and 15 females, while in the research group, the age was 35–76 years old, with an average of (50.96 ± 3.65) years, including 16 males and 14 females. There was no statistical significance in the general data of the two groups. This study was permitted by the Medical Ethics Association of our hospital, and all patients noticed informed consent. This is a prospective study, and the baseline data of the two groups are balanced.

Inclusion criteria are as follows: (1) diagnostic criteria of TBI: clear history of TBI, confirmed by CT or MRI, and accorded with the diagnostic criteria of brain injury approved by the fourth National Academic Conference on Cerebrovascular Diseases; (2) age more than 14 years old and less than 60 years old and graduated primary school education level or above; (3) Glasgow coma scale (GCS) score ≤ 8; (4) Montreal Cognitive Assessment (MoCA) scale < 26 points; (5) no previous history of cognitive impairment and mental illness; and (6) informed consent.

Exclusion criteria are as follows: (1) age ≥60 years old or ≤14 years old and the educational level below primary school; (2) unstable vital signs; (3) MoCA > 26 points; (4) patients with severe language impairment, severe vision or hearing impairment, and bilateral upper limb dysfunction who could not be tested; (5) patients with the previous history of mental illness and pre-TBI with cognitive impairment or suspected cognitive impairment; (6) patients with myocardial infarction or severe hepatic and renal dysfunction, severe infection, and severe diabetes; and (7) unconscious.

### 2.2. Treatment Methods

The control group received early rehabilitation nursing mode and carried out rehabilitation training with joint stability training as the core under the guidance of rehabilitation doctors and nurses in our hospital. First of all, nurses, family nurses, and patients were trained on the relevant knowledge of joint stability training of brain trauma, and rehabilitation doctors conducted rehabilitation training regularly with the participation of the three parties. The stability rehabilitation training program mainly includes antimuscle contracture training, flexion and extension muscle strength training, walking training, and joint impedance training. Meanwhile, rehabilitation doctors record the specific contents of stability training as videos and distribute them to patients for learning. Training is conducted twice a day for 60 minutes each time. The training process is carried out under the guidance of the rehabilitation therapist. It should be noted that attention should be paid to the safety risks in the rehabilitation process to ensure the quality and safety of rehabilitation training.

The research group accepted the early rehabilitation nursing model based on Orem's self-care theory: the Orem self-care model was employed throughout the whole nursing process of the intervention group. According to the patients' health needs, conditions, and existing nursing problems, the patients' self-care ability was comprehensively judged and dynamically evaluated, and different nursing systems and health education were given to guide the patients' self-care theory. Explain the significance and purpose of self-care and the responsibilities that patients and medical staff should bear in the process of rehabilitation training. And adjust flexibly according to the specific condition in the nursing process; the specific nursing process is as follows: (1) diagnosis and treatment: collect data by observing and talking with patients and their families and evaluate the patient's self-care needs, self-care ability, and self-care defects to determine why the patient needs care, and this analysis should be carried out continuously from the beginning to the end. In this step of the investigation, nurses should answer the following five questions: (1) what are the current therapeutic self-care needs of patients? (2) Are there any deficiencies in patients' self-care ability in order to meet these therapeutic self-care needs? (3) If there is a self-protection defect, what is the nature and why? (4) In order to achieve the purpose of treatment, should patients be helped to complete all their work or those self-care abilities that have been developed should be protected and encouraged? (5) What is the potential of patients' self-care ability in the future, such as increasing or deepening self-care knowledge, learning nursing techniques, and cultivating self-care desire? How to effectively and continuously integrate major self-care measures into daily life and self-care plans. (2) Planning and design: this step is the planning part of the procedure. According to the nursing diagnosis, the corresponding nursing plan is designed according to the three systems of complete compensation, partial compensation, and auxiliary education in the nursing system theory. The specific plans are as follows: (1) complete compensation nursing: the patients are not fully awake after joint replacement surgery, the affected limbs use brace, traction and other braking, drainage tubes, catheters, and so on, and the patients are unable to take care of themselves, their own hygiene, and daily life, and concomitantly, we should use complete compensation nursing system nursing with medical support to compensate for patients' lack of self-care. Nurses' behavior is to help them accept compensation and provide for all their self-care needs, including condition observation, cleaning respiratory tract, nutrition, keeping incision dry, keeping drainage tube unobstructed, observing local inflammation, recording body temperature changes, and basic life care such as washing, eating, defecation, cleaning perineum, skin care, medication, and so on. At the same time, it can meet the therapeutic self-care needs of patients, including helping patients turn over, massage the compressed site, measure vital signs, keep the indwelling catheter unobstructed, observe drainage, and so on. (2) Partial compensatory nursing: when the patient's vital signs are stable, conscious, and pain relieved, it generally refers to the second day after operation to before discharge. Nurses should continue to evaluate the patient's physical condition, postoperative rehabilitation exercise knowledge, skills, and self-care ability and provide partial compensatory nursing according to the difference in self-care ability. Let patients do what they can, change passivity into the initiative, mobilize patients' subjective initiative, and enhance patients' self-care ability and behavior ability, with emphasis on assistance, guidance, and education. During this period, the main goal of nursing is to teach patients specific skills of self-care and functional exercise. The functional exercise program and the training procedure for patients' self-care ability were formulated and photocopied and distributed to the patients themselves or their families, which were guided by the researchers. In the process of implementation, according to the specific conditions of each patient, adjust the exercise intensity and progress and help patients answer common questions in postoperative rehabilitation. (3) Support education and nursing: nurses use the support-education system to give psychological support to patients, provide counseling, carry out health education and nursing behavior guidance, and guide patients and their families to participate actively. Patients with brain trauma have different degrees of lack of knowledge in each rehabilitation stage. Nurses, as health promoters and educators, should provide relevant information and health education to patients and their families in the whole process of the disease. Make them master the methods of self-care, guide patients on how to prevent dislocation, let patients and their families actively participate in nursing activities, and achieve the goal of restoring independent living ability as soon as possible, including making the introduction of admission to the hospital; making the patients familiar with the ward environment and medical staff as soon as possible; reducing depression; talking with patients before operation; patiently introducing the basic knowledge of brain trauma, including the basic principle and treatment effect; providing relevant information to patients; comforting and encouraging patients with successful examples of surgery; increasing treatment confidence; relaxing their spirit; eliminating worries and fears; and actively cooperating with medical care. After the operation, most patients may have fear and pain when using a venous pump and joint activity. Researchers should patiently explain to this kind of patients, eliminate their fear and anxiety, and encourage patients to build up confidence in overcoming the disease. (3) Implementation and evaluation: this step is the generation and management of the nursing relationship. Nurses implement the nursing system plan. Orem believes that nurses should: (1) assist patients (or family members) in self-care to achieve healthy results, (2) check completion against the requirements of the plan, (3) collect evidence that can show the effectiveness of nursing, and (4) compare with the results determined in the nursing system and evaluate the results with evidence.

### 2.3. Observation Index

#### 2.3.1. Satisfaction

After consulting the literature and expert discussion, we designed patients' follow-up satisfaction, a total of 10 items, and recorded patients' satisfaction with follow-up management mode, health education, medical and nursing service, appointment registration process, and so on. It is assigned into four dimensions: very satisfied, satisfied, general, and dissatisfied. Satisfaction rate = very satisfaction rate + satisfaction rate + general rate.

#### 2.3.2. Fugl-Meyer Motor Function Scale

Fugl-Meyer's motor function scale was used to evaluate the upper limb motor function and lower limb motor function before nursing and 1 month, 2 months, and 3 months after nursing. The upper limb motor function score was 66, and the lower limb motor function score was 34. The higher the score, the better the patient's motor function.

#### 2.3.3. NIH-SS Scoring

The neurological function of the patients was evaluated with NIH-SS score before nursing and 1 month, 2 months, and 3 months after nursing, including 11 items such as level of consciousness, gaze, visual field, facial paralysis, upper limb movement, lower limb movement, ataxia, sensation, language, dysarthria, and neglect. The higher the score, the more obvious the neurological dysfunction.

#### 2.3.4. Barthel Index

Before nursing and 1 month, 2 months, and 3 months after nursing, the Barthel index was employed to evaluate the ability of daily living before and after the intervention. The total score was 100. The higher the score, the stronger the ability to daily living.

### 2.4. Quality of Life Scale

The quality of life scale consists of four subscales, including physical, psychological, social, and health self-awareness, with a total of 29 items. Cronbach's *α* coefficient of the scale is 0.79–0.91. The scale was scored by 1–5 grades. The lower the score, the higher the quality of life.

### 2.5. Compliance

Compliance is assigned into very compliance, compliance, noncompliance, and compliance rate = very compliance rate + compliance rate.

### 2.6. Statistical Analysis

The data were analyzed by SPSS 21.0 statistical software, and the measurement data were expressed by ($\bar{x}$ ± *s*). *T*-test of independent samples was employed for comparison between groups; paired *t*-test was employed for comparison before and after treatment; and counting data were expressed by example *n* (%). *χ*^2^ test was employed; *P* < 0.05 exhibited the difference was statistically significant.

## 3. Results

### 3.1. Comparison of Nursing Satisfaction

**Figure 1:**
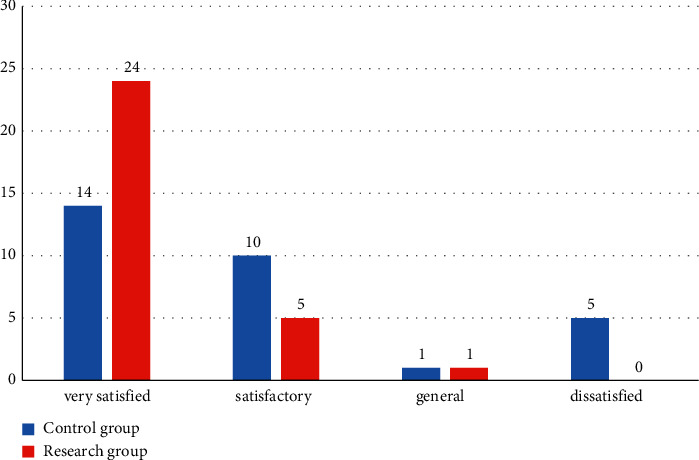
Comparison of nursing satisfaction between the two groups.

First of all, we compared the nursing satisfaction between the two groups: the research group was very satisfied in 24 cases, satisfactory in 5 cases and general in 1 case; the satisfaction rate was 100.00%; the control group was very satisfied in 14 cases, satisfactory in 10 cases, general in 1 case, and dissatisfied in 5 cases. The satisfaction rate was 83.33%. In the comparison of groups, the nursing satisfaction in the research group was higher, and the difference was statistically significant (*P* < 0.05). All the data results are indicated in Figure 1.

### 3.2. Fugl-Meyer Score Comparison

Secondly, we compared the Fugl-Meyer scores of the two groups. Before nursing, there exhibited no significant difference (*P* > 0.05). After nursing, the Fugl-Meyer score of both groups increased. Compared with the control group, the Fugl-Meyer scores of the research group were higher at 1 month, 2 months, and 3 months after nursing, and the difference was statistically significant (*P* < 0.05). All the data results are indicated in Table 1.

### 3.3. NIH-SS Score Comparison

Thirdly, we compared the NIH-SS scores of the two groups. Before nursing, there exhibited no significant difference (*P* > 0.05). After nursing, the NIH-SS scores of both groups decreased. Compared with the control group, the NIH-SS score of the research group was lower at 1 month, 2 months, and 3 months after nursing, and the difference was statistically significant (*P* < 0.05). All the data results are indicated in Table 2.

### 3.4. Barthel Index Comparison

Then, we compared the Barthel index. Before nursing, there exhibited no significant difference (*P* > 0.05). After nursing, the Barthel index of the two groups increased. The Barthel index of the research group was higher compared to the control group at 1 month, 2 months, and 3 months after nursing, and the difference exhibited statistically significant (*P* < 0.05). All the data results are indicated in Table 3.

### 3.5. Comparison of Qualities of Life Scores

Next, we compared the scores of qualities of life between the two groups. Before nursing, there exhibited no significant difference (*P* > 0.05). After nursing, the scores of qualities of life of the two groups decreased. The scores of physiological function, psychological function, social function, and health self-cognition in the research group were lower than those in the control group, and the difference was statistically significant (*P* < 0.05). All the data results are indicated in Table 4.

### 3.6. Compliance Comparison

**Figure 2:**
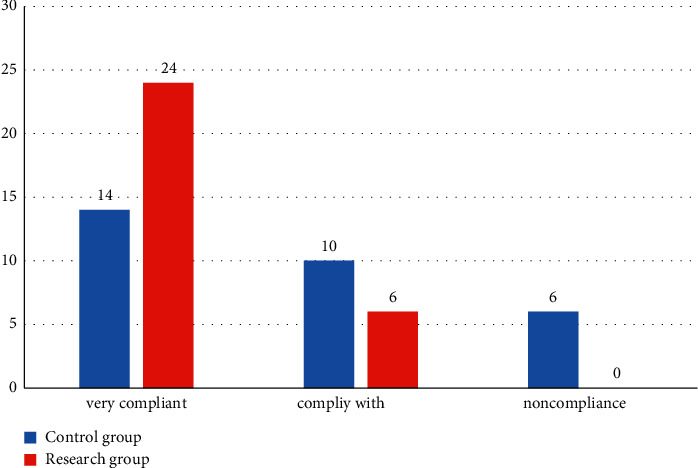
Comparison of compliance between the two groups.

Finally, we compared the compliance of the two groups; the compliance rate of the research group was higher; and the difference was statistically significant (*P* < 0.05). All the data results are indicated in Figure 2.

## 4. Discussion

TBI refers to the brain injury caused by external violence, which can lead to disturbance of consciousness, memory loss, and neurological dysfunction [13]. Studies have indicated that TBI has the characteristics of high morbidity, high mortality, and high disability rate [14]. Craniocerebral injury is the most common and crippling disease in nervous system diseases [15]. Patients may have varying degrees of motor and sensory dysfunction, accompanied by recognition, cognitive and perceptual functions, language communication functions, ability to take care of daily life, and behavioral, psychological, and social communication disorders; these dysfunctions lead to high disability rates and seriously affect the quality of life of patients [16]. It brings great influence and a heavy burden to the individual, family, and society of the patients. With the rapid development of transportation, the incidence of TBI is getting higher [17]. Today, with the rapid development of medical technology, great progress has been made in the treatment of TBI [18]. The overall mortality rate of TBI has decreased from 50% 30 years ago to about 30% at present, and the case fatality rate has dropped obviously [19]. However, as people often attach importance to life rescue while neglecting functional rehabilitation, the disability rate is greatly increased, and most of the surviving patients will leave behind varying degrees of disability. In addition to cognitive dysfunction, language dysfunction, and physical function accounted for a large part [20]. In the United States, some scholars published the meta-analysis report “early rehabilitation based on evidence: recommendations for Clinical practice” from 1999 to 2000. The results support the effectiveness of existing early rehabilitation methods for patients with stroke and TBI [21]. The evidence demonstrates that perceptual rehabilitation training after stroke in the left and right hemispheres and rehabilitation training of attention, memory, and executive dysfunction after TBI contribute to functional improvement [22]. In 2005, American scholars systematically reviewed the literature on the efficacy of limb rehabilitation in patients with acquired brain injury published from 1998 to 2002 and put forward more specific and detailed practical standards, guidelines, and choices [23]. Other scholars' studies performed meta-analysis, which also indicated that the therapeutic effect was affected by the type of cognitive impairment, course of the disease, type of TBI, and age and indicated sufficient evidence to confirm the effectiveness of attention training after TBI and visual-spatial training after unilateral neglect after stroke [24]. In China, as people pay more attention to limb functional rehabilitation, with the development of limb functional rehabilitation in Europe and the United States, and other developed countries, people gradually realize the impact of limb functional rehabilitation on rehabilitation. Limb functional rehabilitation has been paid more and more attention and become the focus [24].

After TBI, due to the mechanical damage of brain tissue caused by external forces, coupled with the secondary brain inflammatory response, it is more likely to bring about extensive nerve fiber damage, and the damage to brain tissue is more extensive [25]. Therefore, in terms of cerebrovascular disease, the brain is malleable, and the structure and function of the cerebral cortex can change as expected under the continuous action of external stimuli. After TBI, through the considerable intensity of learning and training, the dendrites and axons of neurons can establish new connections, or adjacent intact brain regions can replace the function of damaged brain regions, thus promoting behavioral changes [25]. Therefore, in the early stage of TBI, repeated rehabilitation training can promote the activation of latent pathways and dormant synapses, make the axons of related nerve cells sprout to form new synapses, and finally trigger synaptic regeneration, promoting brain function to carry out the functional repair, so as to reduce the degree of neurological disability. At present, the commonly used clinical cognitive rehabilitation methods include occupational therapy, computer-assisted cognitive training, virtual technology, and remote cognitive rehabilitation [26]. As the ability of TBI patients to process available information is weaker than that of normal people, effective psychological and behavioral patterns should be organically combined in the process of rehabilitation to broaden the information processing and information absorption capabilities of TBI patients, which is also very important to help TBI patients. Therefore, early rehabilitation nursing for patients with TBI is carried out by nurses. The cognitive rehabilitation intervention with the participation of patients' family members and therapists organically combines the mental and behavior patterns of patients. Through repeated training, patients can synthesize, and analyze information, so as to enhance their quality of life [27].

Orem's self-care theory was originally put forward by American nursing theorist Dorothea Orem [28]. Self-care is an individual's self-care behavior to meet the maintenance of their own life, health function, growth, development, and happiness, which is realized by meeting the existing self-care needs. In recent years, there have been more researches and applications on self-care behavior, which have developed rapidly, mainly focused on chronic diseases such as chronic obstructive pulmonary disease, asthma, heart failure, diabetes, hypertension, hemiplegia, and so on [28]. The results show that through self-nursing intervention, patients can master the relevant knowledge of TBI, correctly understand their self-worth, adapt to various changes caused by diseases, thus actively participate in the rehabilitation of TBI, and finally improve their quality of the patient. Orem's self-care theory emphasizes the individual's self-care ability, which is consistent with the holistic nursing concept of “patient-centered.” Its purpose is to facilitate the individual's health status and the body's coping ability and function and has gradually become one of the main means of disease management [29]. The early rehabilitation nursing based on Orem's self-care theory was applied to the nursing of patients with TBI. According to the specific patients and different stages of the disease, the self-care ability was dynamically evaluated, and different nursing systems were adopted. The self-care model suitable for different individuals and stages was worked out so that the nursing care of nursing patients at all levels was valued and compensated according to their self-care ability [30]. It can enable patients to correctly understand the disease and master the relevant self-care knowledge, so as to reduce the occurrence of complications, promote the limb function and survival ability of patients, enhance their self-confidence, and enable them to correctly deal with and accept the changes of internal and external environments, and show better social adaptability and enhance the quality of life. Patients with TBI participate in their own health decision-making and nursing from admission to discharge, encourage patients and their families to participate in nursing activities, mobilize patients' enthusiasm, explore their self-care potential, and give full play to their subjective initiative. It reflects the patient's own value and gives full play to the patient's self-care ability. At the same time, it shortens the nurse-patient relationship, enhances job satisfaction, enhances communication and understanding between nurses and patients, and promotes the harmonious development of the nurse-patient relationship [31, 32]. The same idea can be found in the study put forward by Zhiying Yang et al. [33], who have applied new methods in the study, and the conclusions drawn can also give some support to this study. There are some limitations in this study. First, the sample size of this study is not large, and it is a single-center study, so bias is inevitable. In future research, we will carry out multicenter, large-sample prospective studies, or more valuable conclusions can be drawn.

In conclusion, early rehabilitation nursing for TBI patients based on Orem's self-care theory can effectively improve patient satisfaction and compliance and achieve the purpose of enhancing motor function and living ability. The nursing scheme is worth popularizing in the clinic [34].

## Figures and Tables

**Table 1:** Comparison of Fugl-Meyer score between the two groups[$\bar{x}$ ± *s*, points].

Grouping	*N*	Before nursing	1 month after nursing	2 months after nursing	3 months after nursing
Control group	30	31.96 ± 4.53	44.06 ± 3.12	54.91 ± 2.41	62.59 ± 3.46
Research group	30	31.82 ± 3.56	57.93 ± 2.56	65.18 ± 4.23	74.81 ± 3.13
*t*		0.133	**18.823**	**11.554**	**14.345**
*P*		＞0.05	＜0.01	＜0.01	＜0.01

**Table 2:** Comparison of NIH-SS scores between the two groups[$\bar{x}$ ±* s*,points].

Grouping	*N*	Before nursing	1 month after nursing	2 months after nursing	3 months after nursing
Control group	30	34.91 ± 3.31	32.49 ± 1.55	28.59 ± 4.12	25.78 ± 3.31
Research group	30	34.78 ± 3.56	30.18 ± 3.13	25.44 ± 3.56	20.64 ± 3.13
*t*		0.146	**3.622**	**3.168**	**6.179**
*P*		＞0.05	＜0.01	＜0.01	＜0.01

**Table 3:** Comparison of Barthel index between the two groups[$\bar{x}$ ± *s,* points].

Grouping	*N*	Before nursing	1 month after nursing	2 months after nursing	3 months after nursing
Control group	30	30.68 ± 4.52	34.78 ± 3.12	40.81 ± 3.55	58.49 ± 3.12
Research group	30	30.85 ± 3.77	38.93 ± 5.23	47.49 ± 4.78	65.81 ± 3.45
*t*		0.158	**3.732**	**6.145**	**8.619**
*P*		＞0.05	＜0.01	＜0.01	＜0.01

**Table 4:** Comparison of quality of life scores between the two groups[$\bar{x}$ ± *s*, Points].

Grouping	*N*	Physiological function		Psychological function		Social function		Healthy self-cognition	
		Before nursing	After nursing	Before nursing	After nursing	Before nursing	After nursing	Before nursing	After nursing
Control group	30	15.64 ± 4.91	13.86 ± 2.56^a^	16.31 ± 3.88	14.78 ± 4.31^a^	18.75 ± 3.31	16.23 ± 2.12^a^	15.88 ± 3.78	13.12 ± 1.67^a^
Research group	30	15.41 ± 4.67	11.54 ± 2.31^b^	16.74 ± 3.13	12.88 ± 1.21^b^	18.55 ± 3.76	12.78 ± 3.31^b^	15.12 ± 3.77	10.12 ± 2.77^b^
*t*		0.185	3.682	0.472	2.324	0.218	4.807	0.779	5.080
*P*		＞0.05	＜0.01	＞0.05	＜0.01	＞0.05	＜0.01	＞0.05	＜0.01

*Note*. The control group before and after nursing, ^a^*P* < 0.05; the research group before and after nursing, ^b^*P* < 0.05.

## Data Availability

The data sets used and analyzed during the current study are available from the corresponding author upon reasonable request.

## References

[1] Liu Y., Ma D. (2021). Effect of crisis management intervention combined with hyperbaric oxygen therapy on clinical efficacy, neurological function and prognosis of patients with emergency brain trauma. *Chinese Journal of nautical Medicine and Hyperbaric Medicine*.

[2] Zhao S., Fang P., Tao Y. (2021). Effect of oral nursing intervention on pulmonary infection in patients with brain injury undergoing endotracheal intubation and general anesthesia during operation. *Nursing Research*.

[3] Chen P., Zheng Y., he Z. (2021). One case of planned catheter removal through periodic evaluation in patients with severe brain trauma. *Theory and practice of Rehabilitation in China*.

[4] Dagod G., Roustan J. P., Bringuier-Branchereau B. S. (2021). Effect of a temporary lying position on cerebral hemodynamic and cerebral oxygenation parameters in patients with severe brain trauma. *Acta Neurochirurgica*.

[5] Qi X., Gao F., Zhang Y., Zhou L. (2021). Evaluation of the value and nursing quality of personalized nursing in patients with mild brain trauma. *Minerva Medica*.

[6] Huang H.Yu, Lee C. S., Chiu T. H. (2022). Clinical outcomes and prognostic factors for prolonged mechanical ventilation in patients with acute stroke and brain trauma. *Journal of the Formosan Medical Association*.

[7] Mario F., Mario G., Lara P. (2021). Mismatch between tissue partial oxygen pressure and near-infrared spectroscopy neuromonitoring of tissue respiration in acute brain trauma: the rationale for implementing a multimodal monitoring strategy[J]. *International Journal of Molecular Sciences*.

[8] Radabaugh H., Bonnell J., Schwartz O. (2021). Use of machine learning to Re-assess patterns of multivariate functional recovery after fluid percussion injury: operation brain trauma therapy. *Journal of Neurotrauma*.

[9] Wang H., Guo A. (2020). Study on the Sinicization of mini mental state examination scale and its reliability and validity in patients with TBI. *Nursing Research*.

[10] Koga M., Toda H., Kinoshita M. (2020). Investigation of the impact of preconditioning with lipopolysaccharide on inflammation-induced gene expression in the brain and depression-like behavior in male mice. *Progress in neuro-psychopharmacology & biological psychiatry*.

[11] McCarty M. F., Lerner A. (2020). Nutraceutical induction and mimicry of heme oxygenase activity as a strategy for controlling excitotoxicity in brain trauma and ischemic stroke: focus on oxidative stress. *Expert Review of Neurotherapeutics*.

[12] Pires Rita C., Haldis O., Eszter U. (2020). Xenon treatment after severe TBI improves locomotor outcome, reduces acute neuronal loss and enhances early beneficial neuroinflammation: a randomized, blinded, controlled animal study. *Critical Care*.

[13] Faropoulos K., Makris D., George F. (2020). The value of anti-epileptic therapy as a prophylactic factor for seizures in the management of moderate TBI[J]. *Future Sci. OA*.

[14] Forcione M., Chiarelli A. M., Davies D. J. (2020). Cerebral perfusion and blood–brain barrier assessment in brain trauma using contrast-enhanced near-infrared spectroscopy with indocyanine green: a review. *Journal of Cerebral Blood Flow and Metabolism*.

[15] Seyed Mostafa A., Alireza S., Mohammad Reza E. (2020). Efficacy of high-dose versus low-dose vitamin D supplementation on serum levels of inflammatory factors and mortality rate in severe TBI patients: study protocol for a randomized placebo-controlled trial. *Trials*.

[16] Narapareddy B. R., Narapareddy L., Lin A. (2020). Treatment of depression after TBI: a systematic review focused on pharmacological and neuromodulatory interventions[J]. *Psychosomatics*.

[17] Mitra S., Khatri S. N., Maulik M., Bult-Ito A., Schulte M. (2020). Allosterism of nicotinic acetylcholine receptors: therapeutic potential for neuroinflammation underlying brain trauma and degenerative disorders. *International Journal of Molecular Sciences*.

[18] McMullan Jason T., Ventura A., LeBlanc Dustin P. (2020). Emergency medical services TBI protocols do not reflect brain trauma foundation guidelines.[J]. Prehospital emergency care. *Official Journal of the National Association of EMS Physicians and the National Association of State EMS Directors*.

[19] Khormi Yahya H., Ambikaipakan S., kelly O. C., Zygun D. (2020). Adherence to brain trauma foundation guidelines for intracranial pressure monitoring in severe TBI and the effect on outcome: a population-based study. *Surgical neurology international*.

[20] Giammattei L., Starnoni D., Maduri R. (2020). Implementation of cisternostomy as adjuvant to decompressive craniectomy for the management of severe brain trauma. *Acta Neurochirurgica*.

[21] Chang F., Li H. Z., Zhang S. Y. (2020). Working memory of patients with mild cognitive impairment due to brain trauma based on fNIRS. *Fa Yi Xue Za Zhi*.

[22] Sirena S., Bridget M., Evan W., Villapol S. (2020). Serum amyloid A is expressed in the brain after TBI in a sex-dependent manner. *. Cellular and molecular neurobiology*.

[23] Pieter Francsois F., Andrew J. P., Malcolm B., Bernard S., Smith K. (2020). The utility of the brain trauma evidence to inform paramedic rapid sequence intubation in out-of-hospital stroke. *BMC Emergency Medicine*.

[24] Catherine D., Dumont M., Jean P., Desautels A., Fantini M. L., Montplaisir J. (2020). Sleep-wake disturbances in hospitalized patients with TBI: association with brain trauma but not with an abnormal melatonin circadian rhythm. *. Sleep*.

[25] Watanabe H., Nosova O., Sarkisyan D. (2020). Ipsilesional versus contralesional postural deficits induced by unilateral brain trauma: a side reversal by opioid mechanism. *Brain Communications*.

[26] Joris A., Theressa S. C., Beat A. (2020). TBI enhances the formation of heterotopic ossification around the hip: an animal model study. *. Archives of orthopaedic and trauma surgery*.

[27] Qiu S.-Z., Zheng G.-R., Chen B., Huang J. J., Huang G., Hua H. (2020). Prognostic value of admission serum glucose-phosphate ratio in predicting the 6-month outcome of patients with severe TBI: a retrospective study. *Clinica Chimica Acta*.

[28] Evans V. (2021). Caring for traumatic brain injury patients: Australian nursing perspectives. *Critical Care Nursing Clinics of North America*.

[29] Mortimer D. S., Berg W. (2017). Agitation in patients recovering from traumatic brain injury: nursing management. *Journal of Neuroscience Nursing*.

[30] Qi X. (2018). Application of brain state index and clinical pulmonary infection score in evaluating the prognosis of patients with brain injury coma complicated with pulmonary infection. *Nursing research*.

[31] Goreth G. B. (2017). Pediatric mild traumatic brain injury and population health: an introduction for nursing care providers. *Critical Care Nursing Clinics of North America*.

[32] Bi M., Meng L., Bai L. (2022 May). Effects of comprehensive nursing based on Orem’s self-care theory on symptom improvement and pregnancy outcome in patients with antiphospholipid syndrome: a retrospective cohort study. *Computational and Mathematical Methods in Medicine*.

[33] Yang Z., Shao L., Teng Y. (2022). Evaluation of the efficacy and adverse reactions of mirena combined with hysteroscopic surgery when treating AUB: based on a retrospective cohort study. *Computational and Mathematical Methods in Medicine*.

[34] Castañeda C. S., Marysol S. O., Luis C., Suárez S. A. O., Rocha L. (2020). Propylparaben reduces the long-term consequences in Hippocampus induced by TBI in rats: its implications as therapeutic strategy to prevent neurodegenerative diseases. *Journal of Alzheimer’s disease: JAD*.

